# Association of Iron Depletion with Menstruation and Dietary Intake Indices in Pubertal Girls: The Healthy Growth Study

**DOI:** 10.1155/2013/423263

**Published:** 2013-12-23

**Authors:** George Moschonis, Dimitrios Papandreou, Christina Mavrogianni, Angeliki Giannopoulou, Louisa Damianidi, Pavlos Malindretos, Christos Lionis, George P. Chrousos, Yannis Manios

**Affiliations:** ^1^Department of Nutrition, Harokopio University of Athens, 70 El.Venizelou Avenue, Kallithea, 17671 Athens, Greece; ^2^Department of Public Health and Nutrition, Zayed University, Abu Dhabi 144534, UAE; ^3^Department of Pathology, General Hospital of Volos, 38222 Volos, Greece; ^4^Clinic of Social and Family Medicine, School of Medicine, University of Crete, Heraklion, 71003 Crete, Greece; ^5^First Department of Pediatrics, University of Athens Medical School, Athens, Aghia Sophia Children's Hospital, 11527 Athens, Greece

## Abstract

The aim of the present study was to investigate the associations of iron depletion (ID) with menstrual blood losses, lifestyle, and dietary habits, in pubertal girls. The study sample comprised 1222 girls aged 9–13 years old. Biochemical, anthropometrical, dietary, clinical, and physical activity data were collected. Out of 274 adolescent girls with menses, 33.5% were found to be iron depleted (defined as serum ferritin < 12 **μ**g/L) compared to 15.9% out of 948 girls without menses. Iron-depleted girls without menses were found to have lower consumption of poultry (*P* = 0.017) and higher consumption of fruits (*P* = 0.044) and fast food (*P* = 0.041) compared to their peers having normal iron status. Multivariate logistic regression analysis showed that girls with menses were 2.57 (95% CI: 1.37, 4.81) times more likely of being iron depleted compared to girls with no menses. Iron depletion was found to be associated with high calcium intake, high consumption of fast foods, and low consumption of poultry and fruits. Menses was the only factor that was found to significantly increase the likelihood of ID in these girls. More future research is probably needed in order to better understand the role of diet and menses in iron depletion.

## 1. Introduction

The combination of low dietary iron intake and high iron losses may lead to a depletion of iron stores and consequently to iron deficiency. Iron deficiency is one of the most prevalent nutritional disorders, with the World Health Organization (WHO) estimating a total prevalence of about 66–80% worldwide, particularly in developing countries [1]. Iron deficiency is very common in all age groups but particularly among infants and adolescents [1]. Most of children with iron deficiency show no clinical signs that could provide an indication for an early diagnosis and treatment. In most cases iron deficiency is diagnosed accidentally during routine checkups or due to other medical issues. Iron deficiency that occurs in childhood may have several adverse health effects on the immune function, cognitive development, temperature regulation, and energy metabolism and therefore should be diagnosed early as possible and treated appropriately [2].

Ferritin is an iron-binding protein with its serum levels reflecting iron stores. When serum ferritin (SF) levels drop below the threshold of 15 or 12 *μ*g/L, this is defined as iron depletion (ID), which if not corrected can reduce erythropoiesis, consequently leading to anemia [3]. Although SF is the most frequent biomarker routinely measured to assess iron stores, and in most cases with high diagnostic accuracy [4] as it is an acute-phase protein, it may increase in states of viral or bacterial infections, acute or chronic diseases, and so forth [4]. This requires special attention when interpreting the levels of SF in the assessment of iron status.

Adolescents are at high risk of ID, as a consequence of their increased iron requirements due to accelerated growth velocity [5]. Moreover in pubertal girls increased iron losses due to menstruation elevate the risk of ID [5]. In this context, Hallberg et al. [6] have reported an increased prevalence of ID in pubertal girls that was found to differentiate based on their menstrual status, dietary habits, use of supplements, and oral contraceptives.

Although dietary iron intake is the major source of iron influx in the body, the primary homeostatic mechanism for maintaining iron balance is the absorptive efficiency of the small intestine [7]. However, only a small proportion of dietary iron is available for absorption, which mainly depends on composition of the diet. Heme iron, which constitutes about 30–70% of all iron found in meat, is more absorbable than nonheme iron, which is found in both meat and plant foods. The bioavailability of nonheme iron in meals consumed by populations following western dietary patterns has been reported to range from 5 to 40% [8] and is very much affected by other dietary factors and nutrients that either enhance or inhibit its intestinal absorption. More specifically, high meat consumption and high intake of ascorbic acid increase nonheme iron intestinal absorption, while high intake of calcium, polyphenols, and phytate-rich foods and beverages inhibits its absorption [9].

Considering the erratic dietary habits usually recorded among teenage girls [10] and their increased iron losses due to menstruation, the aim of the present study was to investigate the association of ID with menstrual blood losses and lifestyle habits, such as dietary intake and physical activity, in pubertal girls.

## 2. Methods

### 2.1. Sampling

The Healthy Growth Study was a large scale cross-sectional epidemiologic study in Greece initiated in May 2007. Approval to conduct the study was granted by the Greek Ministry of National Education and the Ethics Committee of Harokopio University of Athens and was conducted in accordance with the ethical standards specified in the 1964 Declaration of Helsinki. The study population was comprised of schoolchildren 9–13 years old, attending the 5th and 6th grades of primary schools located in rural municipalities within the counties of Attica, Aitoloakarnania, Thessaloniki, and Iraklio. The sampling of schools was random, multistage and stratified by parents' educational level and total population of students attending schools within municipalities of these counties, as described in more detail elsewhere [11]. The study population was representative of the 9–13-year-old school children living in the four counties under study. These counties are scattered throughout the Greek territory, covering the northern (i.e., Thessaloniki), central (i.e., Attica), western (i.e., Aitoloakarnania), and southern (i.e., Iraklio, Crete) parts of Greece. This combined with the random, multistage and stratified sampling procedures followed to recruit our sample are indicative of the representativeness of our population. The sampling procedure yielded 77 primary schools, representative of the total number of schools in the counties under study, which responded positively when they were invited to participate in the study. An extended letter explaining the aims of the study and a consent form for taking full measurements were provided to all parents or guardians having a child in these schools. Parents who agreed to the participation of their children in the study had to sign the consent form and provide their contact details. Signed parental consent forms were collected for 2656 out of 4145 children (response rate 64.1%).

### 2.2. Anthropometry and Physical Examination

Participants underwent a physical examination by two trained members of the research team. The protocol and equipment used were the same in all schools. Weight was measured at 08:00 in the morning during months of May and June to the nearest 100 g using a Seca Digital Scale (Seca Alpha, Model 770, Hamburg, Germany), not wearing shoes and with the minimum clothing possible (tops and bottoms). Height was measured to the nearest 0.1 cm using a commercial stadiometer (Leicester Height Measure, Invicta Plastics, Oadby, UK) with the pupil standing barefoot, keeping shoulders in a relaxed position, arms hanging freely, and head aligned in Frankfort horizontal plane. One well-trained and experienced female paediatrician in each prefecture determined pubertal maturation (Tanner stage) after thorough visual inspection of breast development [12]. Finally, each girl was asked by the paediatrician about her menstruation status and age of menarche.

### 2.3. Biochemical Indices

Blood samples were obtained for biochemical and haematological screening tests between 08:30 and 10:30 after a 12 h overnight fast. Detailed instructions were provided during the day prior of blood collection both verbally directed to the children and via written letters addressed to the parents, respectively. Professional staff performed venipunctures, using two types of test tubes, one of which contained EDTA, to obtain a maximum of 10 mL blood. EDTA blood was transferred on the same day of collection to a laboratory located in each one of the four prefectures, where it was analyzed in a CELL-DYN haematological autoanalyser (Abbott Diagnostics, Abbott Park, IL, USA) for red blood cell count (RBC), haemoglobin, haematocrit, mean corpuscular volume (MCV), mean corpuscular haemoglobin (MCH), and mean corpuscular haemoglobin concentration (MCHC). The remaining blood was collected in plain test tubes for the preparation of serum, which was divided into aliquots and stored at −80°C. When blood collection was completed in Aitoloakarnania, Thessaloniki, and Iraklio, all serum samples were transported in dry ice to the Laboratory of Nutrition and Clinical Dietetics at Harokopio University where biochemical analyses took place. Serum iron and total iron binding capacity (TIBC) were determined by colorimetric assays (Roche Diagnostics SA, Basel, Switzerland). Serum C-reactive protein (CRP) levels were determined using Quantikine ELISA kit (R&D Systems Europe, Ltd., Abingdon, UK). Finally, SF was measured with a chemiluminescence immunoassay (Siemens Healthcare Diagnostics, Tarrytown, USA). ID was defined as SF < 12 *μ*g/L using the following age- and gender-specific thresholds proposed by UNICEF and the WHO [13].

### 2.4. Assessment of Dietary Intake

A semiquantitative Food Frequency Questionnaire (FFQ) was also used to determine children's habitual food consumption. The FFQ included 24 predefined questions on the average consumption frequency during the previous year (never or rarely; times per month, week, or day). To improve the accuracy of food description, standard household measures (cups, tablespoons, etc.) and food models were used to define amounts where appropriate. The data obtained from the analysis of the FFQ records were used to estimate the daily mean consumption frequency of meat, fish, and poultry. Citrus fruits and cereals are all necessary for the assessment of iron bioavailability. No foods or drinks with Fe supplementation were included in the FFQ. The FFQ also included two questions for collecting information on the use of supplements by children. For those children that reported using dietary supplements, information on their brand was collected and the additional amount of nutrient or nutrients provided by the supplement was added in their total dietary intake.

Dietary intake data for determining energy, macro- and micronutrients were obtained for two consecutive weekdays (i.e., Monday and Tuesday) and one weekend day, most preferably Sunday, using the 24 hour recall technique. The 24 h recall is a standard technique used to assess dietary intake during the previous day and can be successfully applied in children older than 9 years old [14]. Specifically, all study participants were asked to describe the type and amount of foods, as well as all beverages consumed during the previous day, provided that it was a “typical” day according to the participant's perception. The term “typical” refers to a day when no special occasions (e.g., parties, celebrations, illness, etc.) that could affect children's food and drink consumption were taking place. If it was not a typical day, then the 24-hour recall was repeated at the same day of the following week. At the end of each 24-hour recall, the interviewers, who were dieticians rigorously trained to minimize the interviewer's effect, reviewed the collected data with the respondent in order to clarify entries, servings, and possibly forgotten foods. Mean daily intakes of energy and nutrients derived from the three 24 h recalls were estimated and used in the current analyses. Food intake data were analyzed using the Nutritionist V diet analysis software (version 2.1, 1999, First Databank, San Bruno, CA, USA), which was extensively amended to include traditional Greek recipes, as described [15].

In order to detect those children that were underreporting their dietary intake, the ratio of daily energy intake to estimated basal metabolic rate was calculated. The basal metabolic rate was assessed according to the equations of Schofield [16], taking into account age, sex, and weight of children. To identify subjects that underreported their dietary intake during the 24-hour recalls the cut-off points of energy intake/basal metabolic rate ratio proposed by Sichert-Hellert et al. [17] were used for boys and girls aged 6–13 years, respectively. Those subjects found to underreport their energy intake (*n* = 331) were excluded from further analysis with regards to 24-hour recall dietary intake data.

### 2.5. Assessment of Physical Activity Levels

Physical activity levels were assessed via waist-mounted pedometers (Yamax SW-200 Digiwalker, Tokyo, Japan) that have been well validated in children. Detailed instructions on the use of the pedometers were provided by the research team to the study participants, who were instructed to wear the pedometers for seven consecutive weekdays, that is, from Monday to Sunday.

### 2.6. Statistical Analysis

Normality of the distribution of continuous variables was tested using the Kolmogorov- Smirnov test. Normally distributed continuous variables were expressed as mean ± standard deviation (s.d.), while nonnormally distributed continuous variables were reported as median (25th, 75th percentile). The significance of the differences between iron-depleted and noniron-depleted girls was tested using Student's *t*-test for normally distributed variables and Mann-Whitney test for nonnormally distributed variables. To test the effect of the independent variables that were found to be associated at a univariate level with ID, a multivariate logistic regression analysis was conducted and adjusted odds ratios (OR) with 95% confidence intervals (CI) were computed. All reported *P* values refer to two-sided tests. The level of statistical significance was set at *P* < 0.05. Statistical analysis was conducted using the SPSS v. 17.0 software (SPSS Inc., Texas, USA).

## 3. Results

The differences between girls with and without menses regarding ID prevalence are presented in Table 1. One-third (33.5%) of adolescent girls was found to be iron depleted in the menstruating group, compared to 15.9% in the nonmenstruating group (*P* < 0.001).

Table 2 summarizes the differences in normally distributed hematological, biochemical, physical activity and dietary intake indices between girls with normal iron status and ID, with or without menses. Iron-depleted girls with menses were found to have significantly lower levels of hemoglobin (*P* = 0.001), haematocrit (*P* = 0.001), MCHC (*P* = 0.029), RBC (*P* = 0.048), and serum iron (*P* = 0.005), compared to girls with normal iron status and menses. Iron-depleted girls with menses were found to have significantly higher dietary calcium intake compared to their peers with normal iron status (*P* = 0.033). Iron-depleted girls with menses also had lower levels of hemoglobin (*P* = 0.044), RBC (*P* = 0.037), and SF (*P* = 0.043) compared to iron depleted girls without menses. No other significant differences were observed.

Similarly, Table 3 displays the differences in nonnormally distributed anthropometric (i.e., BMI), hematological, and dietary intake indices between girls with normal iron status and ID, with and without menses. Iron-depleted girls without menses were found to have lower consumption of poultry (*P* = 0.017) and higher consumption of fruits (*P* = 0.044) and fast food (*P* = 0.041) compared to their nonmenstruating counterparts with normal iron status. Furthermore, iron-depleted girls with menses were found to have lower levels of MCV (*P* = 0.015) and higher consumption of packed fruit juice (*P* = 0.048) compared to their peers with normal iron status. Moreover iron-depleted girls with menses were found to be older (*P* < 0.001) and to have higher mean BMI (*P* < 0.001) compared to iron-depleted girls without menses.

The results from the multivariate logistic regression analysis after controlling for girl's age, weight status, and Tanner stage are presented in Table 4. Menstruating girls were found to be more likely for being ID (OR: 2.57; 95% CI: 1.37–4.81) compared to nonmenstruating girls.

## 4. Discussion

The current study showed that the overall prevalence of ID among 9–13-year-old girls in Greece was 24.7%. This prevalence rate is slightly higher than the relevant prevalence reported for Turkish adolescent girls (20.8%) [18]. However, it is much higher than the prevalence reported for girls aged 12–16 years in the US (8.7%) and for adolescent girls in several other European countries that was found to range from 2% to 7% [19]. Furthermore, the current study showed that the prevalence of ID was much higher for girls with menses compared to those without menses (i.e., 33.5% versus 15.9%, *P* < 0.05). Comparable data in adolescent girls from other developed countries is limited; however, there is plenty of evidence available from developing countries. More specifically, lower prevalence rates of ID in girls with menses have been paradoxically observed in rural areas of Nigeria (12.1%) [20], while higher prevalence rates (30.4% and 50%) were observed in Kenyan adolescent and Egyptian college girls, respectively [21, 22]. While one strong risk factor of ID in developing countries is diet poor in iron-rich sources, in girls from both developing and developed countries menstrual blood loss is a very important risk factor for ID [23].

The present study showed that menstrual blood loss was the only factor strongly related to ID in preadolescent girls. When tested at a multivariate level with other significant dietary correlates of ID, it remained positively and strongly associated with ID possibly indicating an independent association. Likewise, other previous studies investigating the association of menstrual blood loss on iron status in adolescent girls have also reported similar findings [8, 20, 21]. Total daily iron requirements in girls almost double during adolescence since they increase from a range of 1.22 to 1.46 mg/day before menarche to a range of 1.39 to 2.54 mg/day after menarche. This is mainly attributed both to the increase of the total blood volume and lean body mass in children entering adolescence and to menstrual iron losses in girls are [24]. In this context the Recommended Dietary Allowance (RDA) for iron also doubles from 8 mg per day in 9–13-year-old girls to 15 mg per day in 14–18-year-old girls [25], following the increase in total body iron requirements.

However, further to the increased requirements and recommended dietary intake of iron in menstruating girls, iron status is also determined by other dietary factors that can affect its bioavailability. In this regard any serious attempt to examine the role of diet on iron status must also take into account those factors that inhibit or enhance dietary iron intestinal absorption and bioavailability [9]. The findings of the present study showed that the iron-depleted girls with menses were found to have a higher intake of dietary calcium and packed fruit juice compared to their peers with normal iron status. In addition, the iron-depleted girls without menses were found to have increased consumption of fast food meals and fruits but lower consumption of poultry compared to similar group girls with normal iron status.

Calcium intake is one of the key nutrients that have been reported to inhibit iron absorption, especially when these two nutrients are ingested simultaneously [26]. The interpretation of this observation could be that higher calcium intake by iron depleted girls may be partly implicated in the aetiology of ID [27]. However, the current study showed that consumption of dairy products (i.e., milk and yogurt), which is the main dietary sources of calcium, did not differentiate significantly between the two groups of iron status (i.e., ID versus normal iron status). Furthermore, the association between calcium intake and ID became insignificant at the multivariate regression analysis, thus leaving menses as the main risk factor for ID.

Fruits and fruit juice could be implicated both in the inhibition or the enhancement of iron bioavailability. Regarding the enhancement of nonheme iron bioavailability by the consumption of these food items, this could be mediated by higher intakes of vitamin C, the concentration of which is considerably high in citrus fruits and fruit juices [28]. On the other hand, the low amount of phytate found in fruits and fruit juices could possibly be as an additional minor factor supporting the inhibitory effect of these food items on dietary iron intestinal absorption. The negative effect of phytates on iron absorption has been reported to be dose dependent and starts at very low concentrations of 2–10 mg per meal [29]. Regarding the negative role of packed fruit juices on the manifestation of ID, perhaps due to their higher content in phytates, their frequent consumption could also be implicated in the aetiology of ID via their direct association with childhood obesity [2]. Along with foods that are prepared in a fast food restaurant and are high in fats and sodium, higher consumption of sugared-sweetened packed fruit juices by children has consistently been associated with adiposity, which has been identified as one of the main risk factors of ID [30]. Although both fast food and packed fruit juice were reported by the present study to be higher in iron-depleted girls compared to their counterparts with normal iron status, when the association of the consumption of these food items with ID was tested at a multivariate level, controlling for girls' weight status, this was not statistically significant.

Meat products such as red meat, poultry, and fish represent excellent dietary sources of highly bioavailable heme iron [9]. Nonetheless, in the present study the consumption of red meat and fish was not found to differentiate significantly between girls with normal iron status and ID. Poultry was the only heme iron-rich food source, the consumption of which was found to be higher in girls with normal iron status compared to girls with ID. In a previous study, Keskin et al. [18] reported higher poultry consumption and lower prevalence of iron deficiency in adolescent Turkish girls of high socioeconomic status compared to their less affluent peers. This observation supports the findings from single-meal radioisotope studies that indicate an enhancing effect of lean meat, such as poultry, on iron absorption compared to vegetarian meals [13]. However, similar to the other food groups poultry consumption was not found to be significantly associated with ID at the multivariate regression analysis.

It is worthy to report that the averaged iron deficient girls were higher in girls who had not reached menses yet. This may be explained by the fact that several factors must be present before iron can be absorbed, such as a healthy digestive system, adequate amounts of certain nutrients, specific enzymes, hormones, and proteins that bind with and carry nutrients into the bloodstream. In addition, serum vitamin B12 and folate must be within the normal range; however, we did not had the opportunity to measure them.

The current study has both strengths and limitations. The Healthy Growth Study was a large scale epidemiological study conducted in a representative sample of children from four prefectures within the wider region of Greece. However, as the study has a cross-sectional design, it is not possible to establish causal relationships about the possible role of menses and diet in the prevalence of ID. Finally, the use of qualitative methods (24 h recalls and FFQ) to assess dietary intake can also be a source of bias. The discrepancy between what is reported as calcium intake and the servings of dairy products consumption is mainly due to the different dietary assessment techniques used in each case (i.e., three 24 h recalls and FFQ, resp.). It is well documented in the literature that one of the main limitations of the FFQ is that it underestimates up to a certain extent actual consumption [31].

In conclusion the present study showed a higher prevalence of ID among girls with menses. Iron depletion was found to be associated with low intake of poultry and fruits and high consumption of calcium and fast foods. Furthermore, menses was the only factor that was found to significantly increase the likelihood of ID in these girls after controlling for potential confounders. More future research is probably needed in order to better understand the role of diet and menses on the manifestation of ID and consequently of the preventive actions that will need to be taken for tackling this public health problem.

## Figures and Tables

**Table 1:** Iron depletion based on serum ferritin (<12 *μ*g/L) in adolescent girls with and without menses.

Iron status	Girls without menses	Girls with menses	*P*
	*n* = 948	*n* = 274	
Normal iron status	797 (84.1%)	182 (66.5%)	0.001
Iron depletion	151 (15.9%)	92 (33.5%)	

All data are presented as frequencies and proportions (%).

*Derived from Pearson's chi-square test.

**Table 2:** Differences in hematological, biochemical, dietary, and physical activity indices between girls with normal iron status and iron depletion (serum ferritin < 12 *μ*g/L), with or without menses.

	Girls without menses			Girls with menses			*P* value^§^
	Normal iron status (*n* = 797)	Iron depletion (*n* = 151)	*P* value^‡^	Normal iron status (*n* = 182)	Iron depletion (*n* = 92)	*P* value^‡^	
Hematological and biochemical indices							
Hemoglobin (g/dL)	13.1 ± 0.8	13.1 ± 0.7	0.674	13.4 ± 0.9	12.9 ± 0.8	**0.001**	**0.044**
Haematocrit (%)	39.2 ± 2.1	39.2 ± 1.9	0.941	39.7 ± 2.4	38.7 ± 2.1	**0.001**	0.058
MCHC (g/dL)	33.4 ± 1.2	33.5 ± 1.0	0.561	33.7 ± 1.2	33.4 ± 1.0	**0.029**	0.623
RBC (M/*μ*L)	4.9 ± 0.38	4.8 ± 0.3	0.259	4.8 ± 0.4	4.7 ± 0.3	**0.048**	**0.037**
Serum iron (*μ*g/dL)	85 ± 39	78 ± 40	0.067	93.5 ± 39	79.4 ± 37	**0.005**	0.907
Ferritin (*μ*g/L)	33.9 ± 18	11.9 ± 2.85	**0.001**	29.4 ± 13.7	10.4 ± 3.1	**0.001**	**0.043**
C-reactive protein*(ng/mL)	798 ± 643	624 ± 843	0.870	754 ± 1105	562 ± 646	0.800	0.592
Dietary and physical activity indices^a^	(*n* = 597)	(*n* = 120)		(*n* = 117)	(*n* = 56)		
Total protein (g/day)	69.9 ± 21.5	71.4 ± 21.4	0.499	69.5 ± 21.3	74.2 ± 19.5	0.151	0.451
Calcium (mg/day)	1030 ± 386	1096 ± 423	0.118	1004 ± 345	1131 ± 368	**0.033**	0.294
Sodium (mg/day)	1641 ± 688	1725 ± 698	0.228	1707 ± 681	1700 ± 616	0.947	0.360
Total steps (no of steps/day)	11753 ± 4480	12158 ± 4155	0.280	11823 ± 3860	11604 ± 4170	0.675	0.316

All data are presented as mean ± standard deviation.

^‡^Derived from Student's *t*-test, indicates the statistical significance of the differences between girls with ID and normal iron status.

^§^Derived from Student's *t*-test, indicates the statistical significance of the differences between iron depleted girls with and without menses.

*Parameter was log transformed.

^a^Underreporters were excluded from all analysis concerning dietary data.

MCHC: mean cell hemoglobin concentration; RBC: red blood count.

**Table 3:** Differences in BMI, hematological, and dietary intake indices between girls with normal iron status and iron depletion (serum ferritin < 12 *μ*g/L), with or without menses.

	Girls without menses			Girls with menses			*P* value^§^
	Normal iron status	Iron depletion	*P* value^‡^	Normal iron status	Iron depletion	*P* value^‡^	
	(*n* = 797)	(*n* = 151)		(*n* = 182)	(*n* = 92)		
Age (y)	10.9 (10.5–11.5)	11.2 (10.6–11.7)	0.520	11.6 (11.2–11.9)	11.7 (11.3–12.1)	0.154	**<0.001**
BMI (kg/m^2^)	*z* (0.91–1.07)	*z *(0.95–1.04)	0.607	*z* (−1–1)	*z* (0.96–1.04)	0.541	**<0.001**
Hematological indices							
MCV (fL)	82.0 (79.3–84.4)	82.2 (80.1–84.1)	0.852	83.2 (80.7–85.8)	82.5 (79.6–85.3)	**0.015**	0.287
MCH (pg)	27.5 (26.3–28.6)	27.7 (26.6–28.5)	0.795	28.2 (26.9–29.3)	27.5 (26.5–28.7)	0.892	0.745
*Dietary indices* ^ a^	(*n* = 597)	(*n* = 120)		(*n* = 117)	(*n* = 56)		
Energy and nutrients							
Energy (Kcal/day)	1740.2 (1525.8–2065.9)	1841.4 (1565.5–2196.8)	0.066	1755.4 (1577.7–1989.8)	1819.4 (1625.6–2206.6)	0.414	0.812
Fat (g/day)	81.2 (67.3–100.6)	85.6 (69.3–102.2)	0.306	77.9 (68.2–95.4)	68.1 (82.8–100.8)	0.471	0.594
Fiber (g/day)	12.0 (9.0–16.2)	12.3 (9.8–15.8)	0.543	12.7 (9.1–19.9)	13.5 (8.7–19.5)	0.933	0.559
Iron (mg/day)	9.7 (7.6–12.9)	9.9 (7.6–12.6)	0.788	9.9 (7.6–13.2)	9.9 (7.8–13.0)	0.883	0.861
Magnesium (mg/day)	210.2 (175.3–260.8)	219.2 (187.1–270.8)	0.170	207.6 (172.2–264.1)	218.0 (194.0–265.4)	0.230	0.861
Selenium (mg/day)	0.10 (0.07–0.2)	0.12 (0.1-0.2)	0.120	0.10 (0.1-0.2)	0.12 (0.1-0.2)	0.071	0.305
Zinc (mg/day)	9.2 (7.4–11.3)	9.2 (7.6–11.4)	0.650	9.0 (7.4–11.0)	9.4 (7.7–11.5)	0.349	0.785
Vitamin C (mg/day)	72.9 (37.1–147.8)	70.2 (41.5–128.2)	0.543	70.9 (43.7–145.6)	106.3 (42.5–170.4)	0.529	0.195
Folate (*μ*g/day)	210.1 (142.8–279.5)	207.6 (143.1–303.0)	0.607	209.0 (143.6–294.1)	239.0 (131.3–293.7)	0.704	0.812
Food items							
Dairy products (servings/day)	1.08 (0.75–2.00)	1.14 (0.75–2.53)	0.283	1.08 (0.66–1.50)	1.14 (0.75–2.53)	0.302	0.262
Cereal (servings/day)	0.36 (0.03–1.00)	0.36 (0.03–1.0)	0.657	0.36 (0.07–1.00)	0.25 (0.03–1.00)	0.502	0.732
Cakes/biscuits (servings/day)	0.14 (0.08–0.36)	0.14 (0.08–0.36)	0.838	0.14 (0.03–0.36)	0.14 (0.07–0.36)	0.908	0.473
Red meat (servings/day)	0.36 (0.14–0.36)	0.14 (0.14–0.36)	0.108	0.36 (0.14–0.36)	0.36 (0.14–0.36)	0.224	0.101
Poultry (servings/day)	0.14 (0.14–0.36)	0.11 (0.07–0.13)	**0.017**	0.14 (0.08–0.36)	0.14 (0.08–0.36)	0.914	0.247
Fish (servings/day)	0.14 (0.08–0.14)	0.14 (0.08–0.36)	0.608	0.14 (0.08–0.14)	0.14 (0.08–0.20)	0.674	0.990
Fruits (servings/day)	1.00 (0.36–1.00)	1.00 (0.36–2.50)	**0.044**	1.00 (0.36–2.50)	1.00 (0.36–2.50)	0.625	0.391
Packed fruit juice (servings/day)	0.14 (0.03–0.71)	0.14 (0.03–0.71)	0.970	0.14 (0.03–1.00)	0.36 (0.14–1.00)	**0.048**	0.913
Vegetables (servings/day)	0.36 (0.14–1.00)	0.71 (0.36–1.00)	0.149	0.36 (0.14–1.00)	0.71 (0.30–1.00)	0.213	0.865
Fast food (servings/day)	0.06 (0.02–0.06)	0.08 (0.03–0.08)	**0.041**	0.08 (0.03–0.08)	0.08 (0.03–0.14)	0.696	0.323
Soda (servings/day)	0.03 (0.03–0.14)	0.08 (0.03–0.14)	0.807	0.08 (0.03–0.36)	0.08 (0.03–0.36)	0.338	0.090

All data are presented as median (25th–75th percentile).

^‡^Derived from the nonparametric Mann-Whitney test, indicates the statistical significance of the differences between girls with ID and normal iron status.

^§^Derived from the nonparametric Mann-Whitney test, indicates the statistical significance of the differences between iron-depleted girls with and without menses.

BMI: body mass index; MCV: mean cell volume; MCH: mean corpuscular hemoglobin.

^a^Underreporters were excluded from all analysis concerning dietary data.

**Table 4:** Multivariate logistic regression analysis* examining the association of menstrual status and consumption of certain dietary intake indices with ID in 890^a^ Greek pubertal girls.

Independent variables	Iron depletion (serum ferritin < 12 *μ*g/L)		
	Odds ratio	95% CI	*P* value
Menstrual status			
Girls with menses	1.00		
Girls without menses	**2.57**	**(1.37, 4.81)**	**0.003**
Calcium intake (mg/day):			
<RDA^b^	1.00		
>RDA	1.28	(0.77, 2.15)	0.344
Poultry (servings/day)	0.19	(0.03, 1.02)	0.074
Fruits (servings/day)	0.99	(0.77, 1.28)	0.956
Packed fruit juice (servings/day)	0.97	(0.67, 1.40)	0.859
Fast food (servings/day)	1.16	(0.14, 1.84)	0.163

*Adjusted for girl's age, weight status, and tanner stage.

^a^Underreporters were excluded from the analysis.

^b^Calcium recommended dietary allowance (RDA) for children 9–13 years old is 1300 mg/day.
